# Supplementary material for: Ferromagnetism in Defected TMD (MoX2, X = S, Se) Monolayer and Its Sustainability under O2, O3, and H2O Gas Exposure: DFT Study
Source: Nanomaterials (Basel). 2023 May 15;13(10):1642. doi: 10.3390/nano13101642 (PMC10221339; doi:10.3390/nano13101642)
Supplement: Supplementary file 1 [file nanomaterials-13-01642-s001.zip › nanomaterials-2336440-supplementary.pdf]

nanomaterials

MDPI

Supplementary Materials

Ferromagnetism in Defected TMD (MoX_2, X = S, Se) Monolayer and Its Sustainability under O_2, O_3, and H_2O Gas Exposure: DFT Study

Anjna Devi [1,2], Neha Dhiman [2], Narender Kumar [2,3,4], Wadha Alfalasi [3,4], Arun Kumar [2], P. K. Ahluwalia [1], Amarjeet Singh [1] and Nacir Tit [3,4,*]

1 Department of Physics, Himachal Pradesh University, Shimla 171005, India; anjnahpu90@gmail.com (A.D.); pk_ahluwalia7@yahoo.com (P.K.A.); amarjeet.sirohi@gmail.com (A.S.)
2 Department of Physics, Swami Vivekanand Government College, Shimla-Kangra Rd, Ghumarwin, Himachal Pradesh 174021, India; 560dhiman@gmail.com (N.D.); bansalnarender25@gmail.com (N.K.); arun242493@yahoo.com (A.K.)
3 Department of Physics, College of Science, United Arab Emirates University, Al-Ain P.O. Box 15551, United Arab Emirates; w_alfalasi@uaeu.ac.ae
4 National Water and Energy Center, United Arab Emirates University, Al-Ain P.O. Box 15551, United Arab Emirates
* Correspondence: ntit@uaeu.ac.ae

Table S1. Geometrical and electronic structure parameters of MoS_2 ML, with and without vacancies. The data include the supercell size ($N_x \times N_y$ primitive cells), number of atoms (N), bond length in the vicinity of the vacancy (b in Å), average formation energy (Eb eV/atom), and bandgap energy (Eg in eV). D and I stand for the direct and indirect bandgap, respectively.

Supercell size, $N_x \times N_y$ (PCs)	Vacancy Type	N (atoms)	Content of Vacancies (%)	b (Å)	$\lvert E_b \rvert$ (eV/atom)	E_g (eV)
1×1	Pristine	3	N/A	2.437[a], 2.41[d]	4.915[a], 5.00[b], 4.890[c]	1.626(D)[a], 1.72(D)[d], 1.80(D)[e], 1.70(D)[f]
4×4	V_{Mo}	47	6.25	2.398	4.726	0.175(D)[a], 0.20(D)[d], 0.22(D)[e]
	V_S	47	3.13	2.421	4.877	0.994(I)[a], 1.09(I)[f]
	V_{S2}	46	6.25	2.423	4.857	0.917(I)[a]
5×5	V_{Mo}	74	4.00	2.397	4.789	0.248(D)[a], 0.20(D)[g]
	V_S	74	2.00	2.420	4.885	1.050(I)[a]
	V_{S2}	73	4.00	2.422	4.873	0.999(I)[a]
6×6	V_{Mo}	107	2.78	2.397	4.823	0.260(D)[a]
	V_S	107	1.39	2.419	4.889	1.079(I)[a]
	V_{S2}	106	2.78	2.421	4.881	1.048(I)[a]
7×7	V_{Mo}	146	2.04	2.397	4.839	0.271(I)[a], 0.28(I)[h]
	V_S	146	1.02	2.419	4.890	1.088(I)[a], 1.17(I)[h]
	V_{S2}	145	2.04	2.420	4.882	1.065(I)[a], 1.13(I)[h]
8×8	V_{Mo}	191	1.56	2.397	4.856	0.279(D)[a]
	V_S	191	0.78	2.419	4.893	1.094(I)[a]
	V_{S2}	190	1.56	2.419	4.890	1.075(I)[a]

[a] Present work; [b] Reference 27, [c] Reference 28, [d] Reference 37; [e] Reference 24; [f] Reference 29; [g] Reference 37; [h] Reference 31.

Table S2. Geometric and electronic structure parameters of $MoSe_2$ ML, with and without vacancies. The data include the supercell size ($N_x \times N_y$primitive cells), number of atoms (N), bond length in the vicinity of the vacancy (b in Å), average formation energy (E_b eV/atom), and bandgap energy (E_g in eV). D and I stand for the direct and indirect bandgap, respectively.

Supercell size, N_x×N_y (PCs)	Vacancy Type	N (atoms)	Content of Vacancies (%)	b (Å)	|E_b| (eV/atom)	M (μ_B)	E_g (eV)
1×1	Pristine	3	N/A	2.560[a], 2.54[b]	4.401[a], 4.53[d], 4.401[e]	0.00[a]	1.460(D)[a], 1.44(D)[b], 1.66(D)[c], 1.44(D)[b]
4×4	V_{Mo}	47	6.25	2.549[a]	4.249[a]	3.99[a], 3.27[b]	0.000(↑)[a], 0.708(D↓)[a], 0.000(↑)[b], 0.708(D↓)[b]
	V_S	47	3.13	2.547[a]	4.384[a]	0.00[a], 0.00[b]	0.998(I)[a], 0.94(I)[b]
	V_{S2}	46	6.25	2.563[a]	4.370[a]	0.00[a]	0.994(D)[a]
5×5	V_{Mo}	74	4.00	2.547[a]	4.305[a]	3.99[a]	0.000(↑)[a], 0.747(I↓)[a]
	V_S	74	2.00	2.546[a]	4.392[a]	0.00[a]	1.031(I)[a]
	V_{S2}	73	4.00	2.561[a]	4.383[a]	0.00[a]	1.047(I)[a]
6×6	V_{Mo}	107	2.78	2.547[a]	4.336[a]	3.99[a]	0.980(I↑)[a], 0.721(D↓)[a]
	V_S	107	1.39	2.546[a]	4.396[a]	0.00[a]	1.037(D)[a]
	V_{S2}	106	2.78	2.560[a]	4.390[a]	0.00[a]	1.050(D)[a]
7×7	V_{Mo}	146	2.04	2.547[a]	4.356[a]	3.99[a]	1.011(I↑)[a], 0.710(I↓)[a]
	V_S	146	1.02	2.545[a]	4.400[a]	0.00[a]	1.037(I)[a]
	V_{S2}	145	2.04	2.557[a]	4.398[a]	0.00[a]	1.051(D)[a]
8×8	V_{Mo}	191	1.56	2.547[a]	4.367[a]	3.99[a]	1.019(I↑)[a], 0.702(I↓)[a]
	V_S	191	0.78	2.543[a]	4.401[a]	0.00[a]	1.035(D)[a]
	V_{S2}	190	1.56	2.556[a]	4.398[a]	0.00[a]	1.052(D)[a]

[a] Present work; [b] Reference 11; [c] Reference 32, [d] Reference 27, [e] Reference 28.

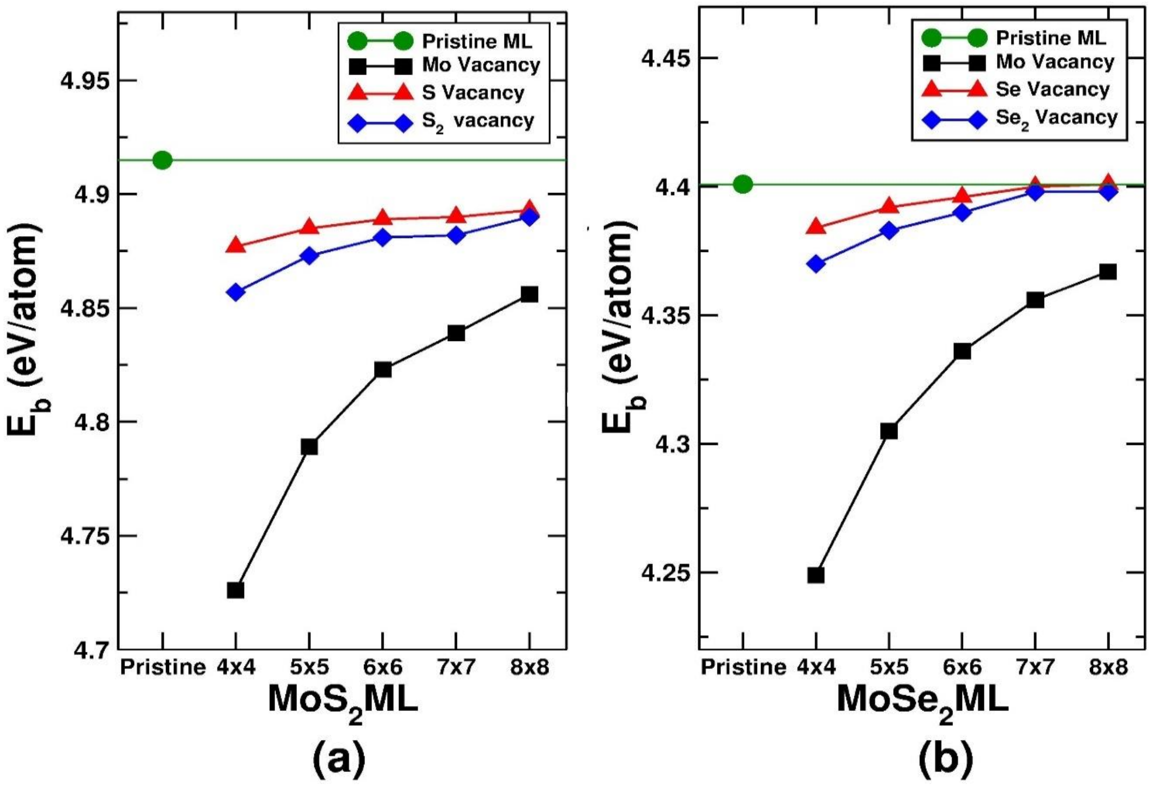

Figure S1. Plot of binding energy strength versus supercell size: (**a**) MoS_2 ML, with and without vacancies; (**b**) $MoSe_2$ ML, with and without vacancies.

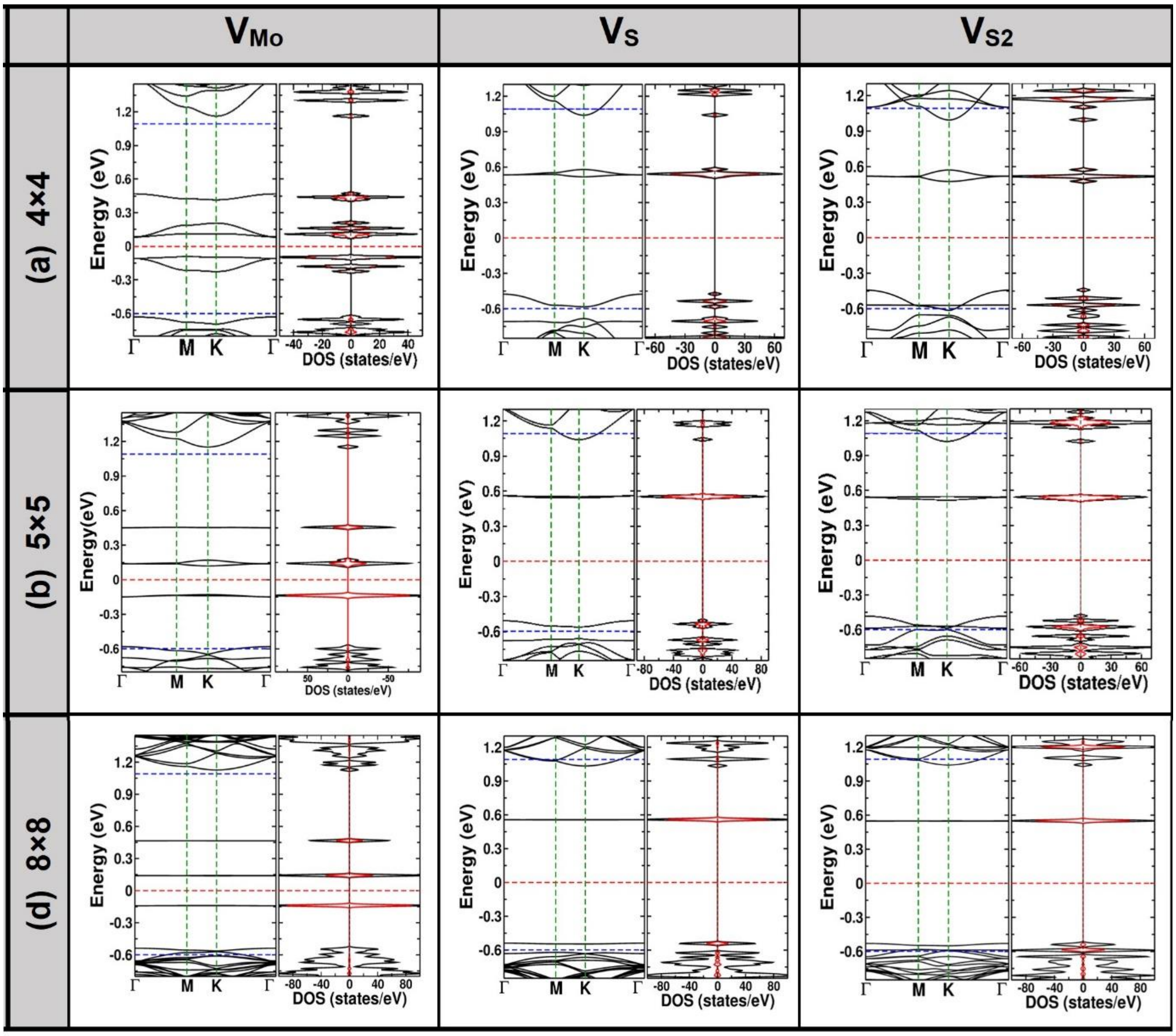

Figure S2. Spin-polarized electronic band structures and density of states (DOS) of vacancy-defected MoS_2 ML versus sample size (i.e., 4 × 4, 5 × 5, and 8 × 8 PCs) and type of vacancy: (**a**) V_{Mo}; (**b**) V_S; and (**c**) V_{S2}. The Fermi energy is chosen as an energy reference (i.e., $E_F = 0$) and is denoted by a red dashed horizontal line. The blue horizontal dashed lines below and above the Fermi level show the valence-band maximum (VBM) and conduction-band minimum (CBM), respectively. In DOS, red and black represent the contributions of atoms near the vacancy and the total DOS, respectively.

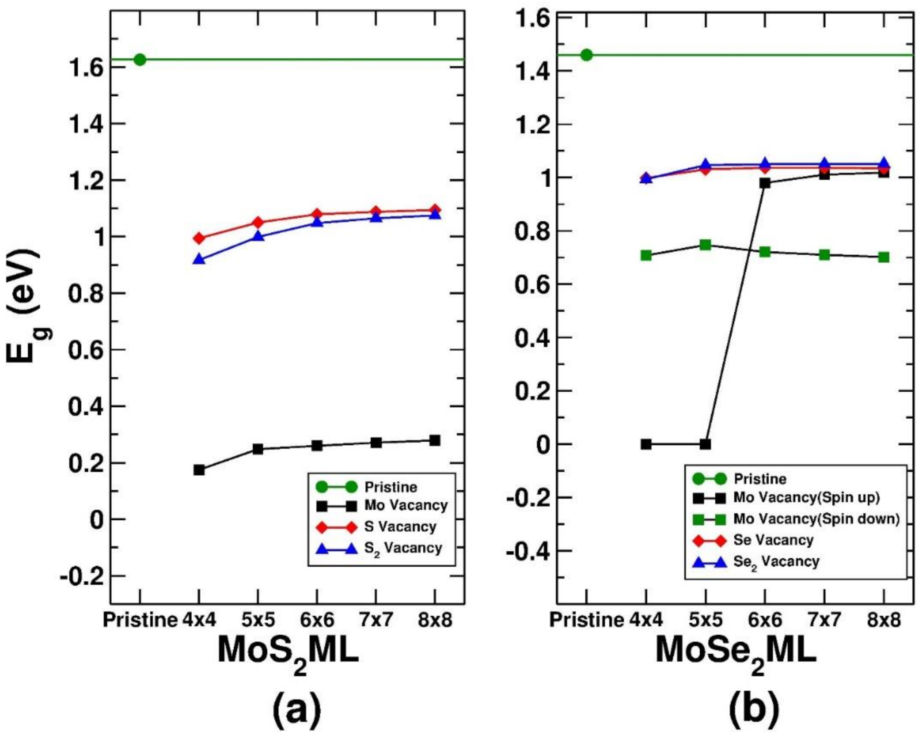

Figure S3. Plots of the bandgap energy (E_g) versus the sample size for MoX_2 ML, with and without vacancies for two cases: (**a**) MoS_2, and (**b**) $MoSe_2$.

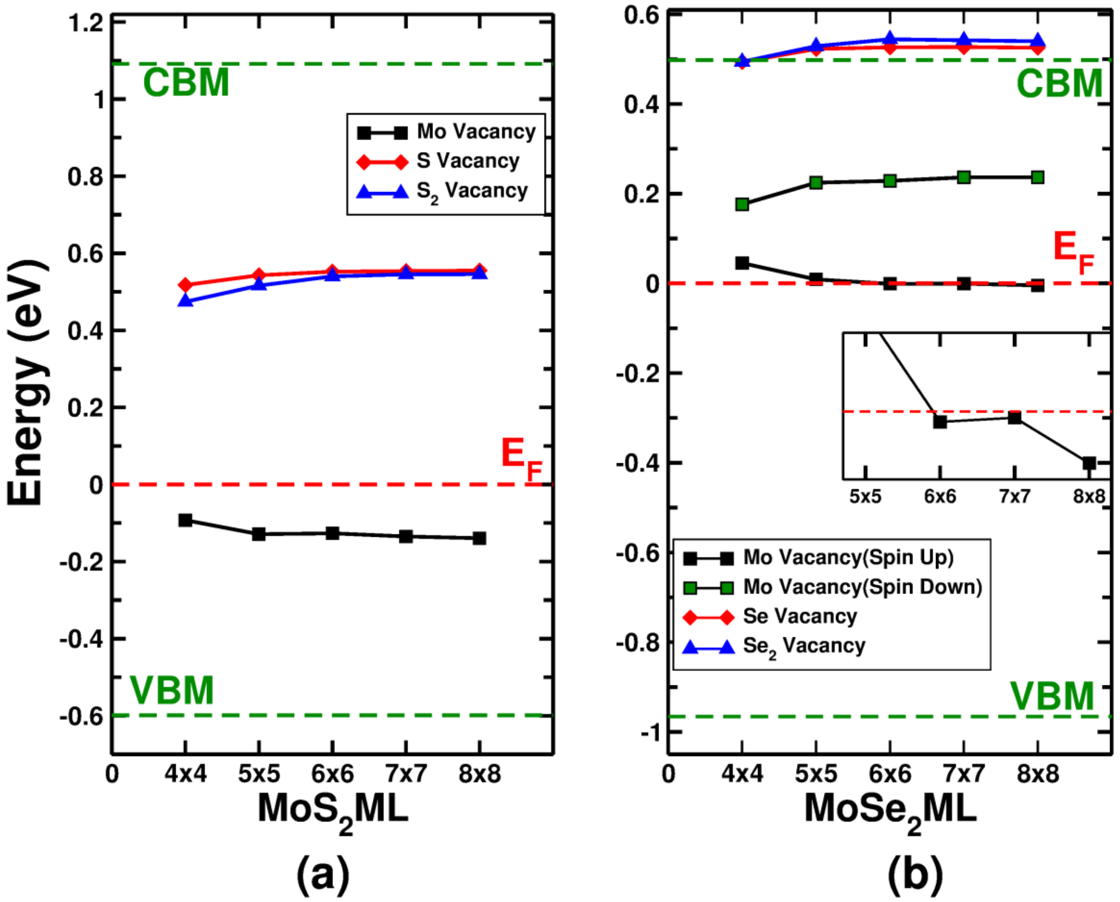

Figure S4. Plots of defect states in both (**a**) MoS_2 ML, and (**b**) $MoSe_2$ ML. The Fermi level is used as an energy reference for all samples and is represented by a red dashed horizontal line. The other two green dashed horizontal lines below and above the Fermi level refer to the VBM and CBM of the pristine structure.
